# Prevalence of Hepatitis B and C Among Healthcare Workers in a Tertiary Care Center in Monrovia Liberia

**DOI:** 10.5334/aogh.3327

**Published:** 2021-07-30

**Authors:** Whitney Lieb, Yassah Moracious Barclay-Korboi, Christopher Dike, Amrin Khander, Samantha Raymond, Tatyana Kushner, Ann Marie Beddoe

**Affiliations:** 1 Division of Global Women’s Health, Department of Obstetrics, Gynecology and Reproductive Science, Icahn School of Medicine at Mount Sinai, New York, NY US; 2 Department of Population Health Science and Policy, Icahn School of Medicine at Mount Sinai, New York, NY, US; 3 Department of Internal Medicine, John F. Kennedy Medical Center, Monrovia, LR; 4 Weill Cornell Division of Maternal-Fetal Medicine, Department of Obstetrics and Gynecology, New York, NY, US; 5 Division of Liver Diseases, Department of Medicine, Icahn School of Medicine at Mount Sinai, New York, NY, US

## Abstract

**Background:**

Viral hepatitis is a leading cause of death worldwide, with a higher mortality rate than that from human immunodeficiency virus (HIV), tuberculosis (TB) or malaria. In order to meet the WHO’s goal of eliminating hepatitis B and C by 2030, there is a dire need to establish baseline prevalence rates and increase public health awareness of this detrimental disease, especially in low and middle-income countries (LMICs) where establishing prevalence rates of hepatitis B (HBV) and hepatitis (HCV) continues to be a problem.

**Objective:**

To establish baseline rates of hepatitis B and C among healthcare workers at the national medical center of Liberia.

**Methods:**

Between October 2017 to 2018 we performed a prospective study to determine rates of hepatitis B and hepatitis C among healthcare workers at John F. Kennedy (JFK) Medical Center, the national medical center of Liberia. All healthcare workers were offered one-time point of care hepatitis B antigen (HBsAg) and hepatitis C antibody testing.

**Findings:**

Two hundred forty-five participants were tested for hepatitis B and C. 15 participants (6.12%) tested positive for hepatitis B [95% CI, 3.47%, 9.90%]. Eleven of the fifteen (73.3%) participants received confirmatory hepatitis B profile testing, and eight (72%) of those were found to be chronic hepatitis B carriers. No participants tested positive for hepatitis C Ab.

**Conclusion:**

Our finding of a greater than 5% prevalence rate, during first line testing, of chronic hepatitis B among health care workers, should help fuel efforts for national testing, vaccination, and treatment efforts in order to align with the WHO goals of elimination of hepatitis B and C by 2030.

## Introduction

Viral hepatitis is a leading cause of death worldwide [1]. The current mortality rate from viral hepatitis is 1.46 million deaths each year, higher than deaths from HIV, TB or malaria. The World Health Organization (WHO) projects that there will be 19 million hepatitis-related deaths between 2015 and 2030, with more than 90% of deaths attributable to complications of hepatitis B (HBV) and C (HCV). Current prevention and treatment methods can reduce the rate of new infections, but the morbidity and mortality of those already infected will continue to rise for years to come [2]. In 2016, the WHO published *Combating Hepatitis B and C to Reach Elimination by 2030*, in which they gave new evidence-based guidelines that recommended the “dramatic scaling up of testing with linkage to care and treatment” of hepatitis infections in low- and middle-income countries [2].

In order to develop programs to help meet the WHO’s goals, there is a need to establish baseline prevalence rates and increase public health awareness, especially in low and middle-income countries (LMICs) where establishing prevalence rates of HBV and HCV continues to be a problem [1,2]. To date, there is limited access to reliable low-cost screening and confirmatory testing in these countries, making accurate estimation rates of HBV and HCV difficult. According to the WHO, worldwide only 9% of HBV-infected persons and 20% of HCV-infected persons have been diagnosed, and only a small number of those people have access to treatment [1]. For example, although West Africa is known to have high prevalence rates of HBV, currently there is no in-country data for HBV or HCV in Liberia [3].

In sub-Saharan Africa, where hepatitis is endemic, most HBV transmission is thought to be from mother-to-child transmission (MTCT)—which occurs during delivery or soon thereafter due to contact with maternal blood and fluid—or close contact between children [4]. In Liberia, prior to the Ebola outbreak, newborn vaccination rates were estimated to be 64–97% [5,6]. However, after the devastation of Ebola and the resultant loss of healthcare infrastructure, that rate dropped to just 27%, and the problem of under-vaccination persists [5,6]. National infant Hepatitis B vaccinations in Liberia restarted in 2016; however, this excluded a large part of the population born during and immediately following the Ebola outbreak that may have been unvaccinated and susceptible to HBV infection. Nevertheless, there is no in-country data to support the projection that Liberia’s rates of infection would surpass that of its neighboring countries [6]. Our study aimed to estimate HBV and HCV prevalence rates among at-risk populations, specifically healthcare workers, at the largest tertiary hospital, John F. Kennedy Medical Center, in Liberia. In a recent paper published by McNaughton et al. found that a test and treat (T&T) vaccination campaign would have a substantial impact on HBV rates [7]. Given these guidelines and those from the WHO, in collaboration with the Liberian Ministry of Health we sought to establish prevalence rates among healthcare workers, a high-risk population and link them to vaccination and care. Knowledge of rates among this at-risk group is a critical starting point to understanding overall country prevalence. Furthermore, we sought to understand transmission patterns by using a high-risk assessment questionnaire in those who were hepatitis B antigen (HBsAg) and HCV antibody positive.

## Methods

### Study design and setting

We performed a prospective study conducted from October 2017 to 2018 to determine rates of HBV and HCV among healthcare workers in Monrovia, Liberia.

This study was conducted at John F. Kennedy (JFK) Medical Center, the national medical center of Liberia. JFK Medical Center is located in the Sinkor district of Monrovia, the capital city of Liberia, with a population of 939,524 as of the 2020 census. With 25% of the total population of Liberia, Monrovia is the country’s most populous city making this a robust sample of the healthcare workforce [8].

In partnership with the Internal Medicine Department at JFK Medical Center, we educated the staff about testing for HBV and HCV. The study was approved by the Institutional Review Board at the Icahn School of Medicine at Mount Sinai and JFK Medical Center.

### Participants

All consecutive healthcare workers throughout the entire hospital were offered one-time point of care hepatitis B antigen (HBsAg) and hepatitis C antibody testing. HIV-1/2 qualitative immunoassay antibody testing was also offered to all participants. Every employee who worked at JFK hospital was offered testing, but no one was required to participate. Announcements explaining the study were made at Grand Rounds and throughout all departments. Testing was offered during the day and night in order to accommodate both daytime and nighttime employees. All participants were informed of their results within two-months. Those who tested positive were referred for confirmatory testing, education, counseling and care options. After confirmatory testing was performed, all participants were connected with internal medicine physicians at JFK Medical Center for either establishment of care or continuity of care and treatment.

For participants who tested positive, a survey was administered to assess possible routes of transmission. This tool was based on the risk assessment tool provided by the US Department of Health and Human Services (HHS), the Centers for Disease Control and Prevention (CDC) and the New York State Department of Health, and modified based on in-country risk factors, including but not limited to Ebola exposure and female circumcision [9,10].

Participants provided consent before any involvement in research. Consenting, screening and confirmation tests were performed by physicians on the study team. All information collected was de-identified to ensure confidentiality, and only available to pre-approved researchers.

### Patient and Public Involvement

Patients and the public were not involved in this research study. The study was performed on healthcare workers and was initiated by a recommendation from the Ministry of Health during the development of the National Cancer Policy, due to concerns of increasing liver cancer cases in Liberia and the need for in-country prevalence data of hepatitis B and C.

### Data collection and management

The questionnaires and test results were de-identified to maintain confidentiality. Results of the screening tests were told only to the participants. Questionnaire data was entered into an encrypted laptop. The data was kept securely in a locked cabinet in Monrovia, Liberia during on-the-ground research. It was then physically transmitted back to the United States and stored in a locked file cabinet in the Principal Investigator’s Mount Sinai School of Medicine office. The de-identified data was then entered into Research Electronic Data Capture (REDCap) 8.10.19 © 2020 Vanderbilt, a secure, web-based software platform hosted at Mount Sinai [11].

### Study procedures

HBV and HCV samples were collected from the participants. HIV testing was additionally offered to all participants. The testing was performed, collected and disposed of by the physicians on the study team after evaluation. Hepatitis B surface antigen (HBsAg) testing was performed using SD Bioline HBsAg WB (whole blood): One Step Hepatitis B Virus Test. Hepatitis B profile testing was offered to all those who tested positive for HBsAg and was performed using SD Hepatitis B Profile test. Hepatitis C testing was performed using OraQuick®—which detects HCV antibodies using saliva or whole blood—or SD Bioline HCV WB antibody test. HIV testing was performed using Alere DETERMINE™ HIV-1/2 and confirmation of HIV status was performed using PCR.

### Statistical analysis

Fisher’s exact tests were used to evaluate differences in prevalence for categorical variables, and Wilcoxon rank sum two-sample tests were used to evaluate differences in continuous variables. Missing categories are displayed for descriptive purposes but were not used in any tests of significance. A logistic regression model—including age, sex and healthcare role—was performed to examine possible predictors of HBV infection. All univariate and multivariate analyses were conducted using SAS software version 9.4 (SAS, Cary, NC 2018).

## Results

Of approximately 300 staff members at JFK Medical Center, 255 individuals volunteered for testing. Seven people declined testing once they read the consent form and three undergraduate university students, non-healthcare workers, were excluded, leaving a total of 245 participants.

Fifteen participants (6.12%) tested positive for hepatitis B during first line testing [95% CI 3.47%, 9.90%]. ***Table 1*** displays HBsAg prevalence by age, sex, role and department. Hepatitis B infection was similar among men and women, (n = 7 [10.29%] and n = 8 [4.65%], respectively; p-value = 0.137). The mean age of HBsAg positive subjects was 38.23 years, similar to the overall population mean of 37.90 years. Hepatitis B infection was also similar among clinical and non-clinical healthcare workers (n = 10 [5.29%] and n = 5 [10.20%], respectively; p-value = 0.201). When looking at risk and healthcare role, medical students and non-clinical staff had the highest rates of HBsAg positivity, 33.3% for each group, making these two populations, 66.7% of the total population of HBsAg positive participants.

**Table 1:** Characteristics of the study population overall and by hepatitis B status.


VARIABLE	ALL (n = 245)	HEPATITIS B NEGATIVE (n = 230)	HEPATITIS B POSITIVE (n = 15)	P-VALUE

Age				0.971

Mean (STD)	37.90 (8.42)	37.88 (8.40)	38.23 (9.09)	

Median (IQR)	37.00 (31.00, 43.00)	37.00 (31.00, 43.00)	37.00 (33.00, 39.00)	

Min and Max	19.00, 60.00	19.00, 60.00	27.00, 58.00	

Missing N (%)	27 (11.02%)	25 (10.87%)	2 (13.33%)	

Sex N (%)				0.137

Female	172 (70.20%)	164 (71.30%)	8 (53.33%)	

Male	68 (27.76%)	61 (26.52%)	7 (46.67%)	

Missing	5 (2.04%)	5 (2.17%)	0 (0.00%)	

Healthcare Role N (%)				0.201

Clinical	189 (77.14%)	179 (77.83%)	10 (66.67%)	

Non-Clinical	49 (20.00%)	44 (19.13%)	5 (33.33%)	

Missing	7 (2.86%)	7 (3.04%)	0 (0.00%)	

Department N (%)				

Emergency Department	2 (0.82%)	2 (0.87%)	0 (0.00%)	

General Staff	90 (36.73%)	81 (35.22%)	9 (60.00%)	

Medicine	34 (15.10%)	35 (15.21%)	2 (13.33%)	

OBGYN/Maternity	49 (20.00%)	48 (20.87%)	1 (6.67%)	

Pediatrics	32 (13.06%)	31 (13.48%)	1 (6.67%)	

Surgery	26 (10.61%)	26 (11.30%)	0 (0.00%)	

Missing	9 (3.67%)	7 (3.04%)	2 (13.33%)	

Specific Healthcare Role N (%)				

Clinical Staff	17 (6.94%)	17 (7.39%)	0 (0.00%)	

Housekeeping	1 (0.41%)	1 (0.43%)	0 (0.00%)	

Intern	1 (0.41%)	1 (0.43%)	0 (0.00%)	

Medical Student	43 (17.55%)	38 (16.52%)	5 (33.33%)	

Midwife	17 (6.94%)	16 (6.96%)	1 (6.67%)	

Non-Clinical Staff	48 (19.59%)	43 (18.70%)	5 (33.33%)	

Nurse	71 (28.98%)	68 (29.57%)	3 (20.00%)	

Physician	40 (16.33%)	39 (16.96%)	1 (6.67%)	

Missing	7 (2.86%)	7 (3.04%)	0 (0.00%)	


P-values do not include missing values. Fisher’s exact tests were used for categorical variables and Wilcoxon two-sample tests for the continuous variable.

**Figure 1:**
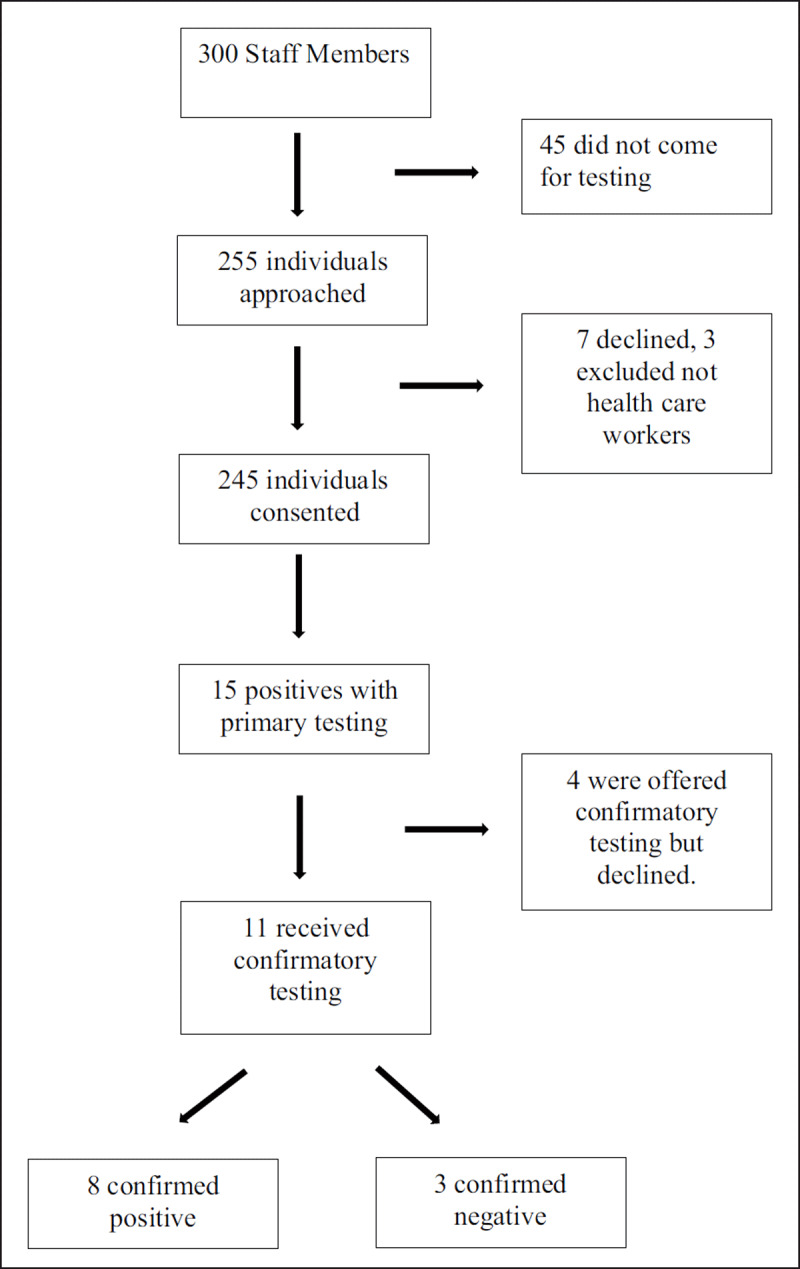
Flow Diagram.

Eleven of the fifteen (73.3%) HBsAg positive participants received confirmation hepatitis B profile testing, and eight (72.7%), were found to be chronic hepatitis B carriers. Two (18.2%) upon confirmatory testing were found to be naïve. And one participant’s profile testing showed a vaccination profile. Therefore, a prevalence rate of 4.89% (12/245) was found of those who underwent confirmatory testing. (See ***Figure 1*** for flow diagram).

Twelve of the fifteen HBsAg positive participants (80.0%) completed the risk assessment tool. ***Table 2*** describes the characteristics of these HBsAg positive participants. Common characteristics found to be associated with positive HBsAg were early age of sexual intercourse (before 19 years old), more than 1 sexual partner in the last year, previously having an STI, and circumcision. Also, 5 (33.3%) reported a family history of liver disease; and 8 (41.7%) stated a history of liver problems but did not acknowledge Hepatitis B infection.

**Table 2:** Characteristics of the hepatitis B positive population.


VARIABLE	ALL (n = 12)

County of Residence	

Bong	2 (16.67%)

Montserrado	9 (75.00%)

Grand Kru	1 (8.33%)

Religion	

Christian	11 (91.67%)

Muslim	1 (8.33%)

Type of Current Work	

Hospital Laundry	1 (8.33%)

Hospital Transport	1 (8.33%)

Medical Records Clerk	1 (8.33%)

Medical Student	4 (33.33%)

Nurse	2 (16.67%)

Sell Groceries	1 (8.33%)

Missing	2 (16.67%)

Education	

High School	2 (16.67%)

Higher Education	9 (75.00%)

None	1 (8.33%)

Stuck with Needle or Sharp	

No	8 (66.67%)

Yes	3 (25.00%)

Missing	1 (8.33%)

Blood in Eyes, Nose, or Mouth	

No	10 (83.33%)

Missing	2 (16.67%)

Ever Received Blood	

No	11 (91.67%)

Yes	1 (8.33%)

Ever Had Surgery	

No	9 (75.00%)

Yes	3 (25.00%)

Ever Had Dental Work	

No	9 (75.00%)

Yes	3 (25.00%)

Liver Problems	

No	7 (58.33%)

Yes	5 (41.67%)

Family History of Liver Disease	

No	8 (66.67%)

Yes	4 (33.33%)

Ebola Survivor	

No	12 (100.00%)

Alcohol Use	

No	8 (66.67%)

Yes	4 (33.33%)

Any Piercings	

No	8 (66.67%)

Yes	4 (33.33%)

Any Tattoos	

No	9 (75.00%)

Yes	3 (25.00%)

Circumcised	

No	2 (16.67%)

Yes	10 (83.33%)

Ever Shared Toothbrush or Razor	

No	9 (75.00%)

Yes	3 (25.00%)

Age First Had Sex	

≤18	7 (58.33%)

>18 Missing	3 (25.00%) 16.67%)

Number of Lifetime Sex Partners	

1–5	6 (50.00%)

6–10	1 (8.33%)

11–15	1 (8.33%)

> 15	3 (25.00%)

Missing	1 (8.33%)

Sex with >1 Person in Last 6 Months	

No	8 (66.67%)

Yes	4 (33.33%)

Ever Had Anal Sex	

No	11 (91.67%)

Missing	1 (8.33%)

Ever Had Sex with Ill Person	

No	10 (83.33%)

Yes	1 (8.33%)

Missing	1 (8.33%)

Ever Had STI	

No	4 (33.33%)

Yes	8 (66.67%)

Ever Tested for HIV	

No	4 (33.33%)

Yes	8 (66.67%)

Tested HIV Positive	

No	8 (66.67%)

Missing	4 (33.33%)

Ever Used Drugs	

No	11 (91.67%)

Missing	1 (8.33%)

Any Sexual Partners Used Drugs	

No	11 (91.67%)

Yes	1 (8.33%)

Ever Injected Unprescribed Drugs	

No	11 (91.67%)

Yes	1 (8.33%)

Even Been in Jail	

No	10 (83.33%)

Yes	1 (8.33%)

Missing	1 (8.33%)


We also performed a logistic regression model including age, sex and role of healthcare role to examine possible predictors of HBV infection (***Table 3***).

**Table 3:** Logistic regression model of hepatitis B status, N = 216.


VARIABLE	UNIVARIATE				MULTIVARIABLE		
	
	OR	95% CI	P-VALUE		OR	95% CI	P-VALUE

Age	1.00	0.94–1.07	0.898		1.00	0.94–1.07	0.871

Sex (Female vs Male)	0.43	0.14–1.35	0.148		0.44	0.14–1.38	0.161

Healthcare Role (Clinical vs Non-Clinical)	0.57	0.17–1.94	0.368		0.61	0.17–2.15	0.444


In logistic regression modeling, we found no association between independent variables (age, sex and healthcare role) and outcome in both our univariate or multivariable models.

Of the 245 participants who were tested for HCV, none were found to be hepatitis C antibody positive. Three participants (1.6%) of the 194 that wanted to be tested for HIV were found to be positive, which was confirmed with PCR testing. All positives were women.

## Discussion

To our knowledge, this is the first study performed of HBV and HCV prevalence in Liberia among healthcare workers. We found a 6.12% prevalence of HBV at first line testing and a 4.89% sample prevalence among cases with available confirmatory testing, 0% of HCV, and 1.6% of HIV among healthcare workers. Medical students and non-clinical healthcare workers had the highest prevalence of HBV.

Previous studies have estimated prevalence rates in Liberia based on surrounding West Africans countries [12]. All current published information is based on extrapolated data from surrounding West African countries. One study from Gower, *et al*., estimates 1.1% of the population in Western sub-Saharan Africa are chronically infected with hepatitis, but this data is based only on previous literature searches [3]. Another study from Mokdad, *et al*., used verbal autopsy data from surrounding countries to estimate mortality rates of liver cirrhosis in Liberia, and surmised it had more than doubled between 1980 and 2010—from approximately 210 to 470 deaths, respectively. That study also calculated the age-standardized mortality rates from liver cirrhosis for both sexes and concluded that it has increased by 6.7% from 1980 to 2010 [12]. However, no actual in-country data was obtained in these studies, or in any studies during our literature search. In-country data is crucial to designing interventions and feasibility projections in order to address the burden of disease and plan a successful public health campaign. Given that JFK is the largest tertiary healthcare center in Liberia, this data gives us insight into rates of infection among healthcare workers.

Countries with prevalence rates of hepatitis B greater than or equal to 8% are considered by the WHO to be high endemic countries, and the WHO recommends introducing the hepatitis B vaccine in these countries’ routine immunization programs [1]. These data should help fuel efforts for vaccination. Our finding that most participants are chronic carriers of hepatitis B is consistent with previous data that suggests most transmission occurs through vertical transmission [1].

That many participants were unaware of their status, and that the prevalence rate was high, points to the importance of future widespread testing, vaccination, and public health efforts to increase awareness and prevention, not only among healthcare workers but also in the general population.

Our finding that medical students and non-clinical staff had the highest prevalence rate of hepatitis B, making up 66.7% of all HBV positive participants, is not unexpected given that these two populations are less likely to be engaged in infection control training in the healthcare setting. Therefore, efforts should be focused on education, prevention, and vaccination specifically targeted to this subpopulation.

We also found that although men made up 27% of study population, they were 46% of the Hep B positive cases, having double the rate of positivity of women, 10.3% vs 4.65%. Given this difference, future interventions should look into possible routes of HBV transmission and susceptibility to infection that may be different among men, and the difference should be confirmed by future prevalence studies.

Our findings of 0% prevalence of hepatitis C is consistent with current data from surrounding countries. A study by Madhava, *et al*. looking at chronic hepatitis C virus in sub-Saharan Africa found that estimated prevalence rates of hepatitis C in West Africa were between 0.0–8.7%, with an average of 2.4%. In Guinea, which neighbors Liberia, the prevalence rate of hepatitis C was found to be 0.8–8.7%, which is in line with our results [13]. Our overall results are further validated by the fact that our HIV prevalence rate of 1.6% is consistent with previous in-country data of 1.6% HIV prevalence rates reported by the WHO [14]. However, it is possible that if we had tested a larger population we could have found a higher prevalence of hepatitis C.

There were several limitations to our study. First, our population was small; however, it did capture a majority of the targeted population, and given that this is a descriptive study, our findings still present important data about the rates of HBV and HCV in this high-risk population. Second, although SD Bioline HBsAg WB One Step Hepatitis B Virus test reports a sensitivity of 100% and specificity of 100%, according to the 2003 WHO Evaluation report [15], our study did not produce the same results. Point-of-care testing sensitivity and specificity was not 100% accurate. This may be due to storage conditions and perhaps unknown damage to point-of-care tests during the supply chain. During our study, two of the HBsAg positive tests using SD Bioline HBsAg One Step Hepatitis B Virus Test were found to have negative results upon confirmatory testing with SD Hepatitis B Profile test, and one patient who tested HBsAg positive was found to test HBsAg negative and HBsAb. Third, four patients who initially tested positive for HBsAg did not present for confirmatory testing, and therefore we were not able to confirm their initial results. Participants were contacted several times and offered free confirmatory testing but declined. Given the stigma of disease, we surmise that participants did not want to follow up to confirm the results. These limitations highlight the difficulty in accessing high quality tests in this region and underscore the challenges to establishing a regional prevalence of disease, as well as linkage to care among those who initially test positive. Given that other infectious diseases such as HIV can be treated based on first line testing without confirmation testing, it is important to highlight the difference between first line testing positives and confirmatory positives when planning future public health projects.

In addition, our results were limited to healthcare workers at JFK Medical Center in Liberia’s capital city; therefore, our conclusions may not extend to rural areas, where healthcare is substantially different. Nonetheless, given Liberia’s limited resources, this in-country study is an important step in beginning to estimate HBV and HCV rates among healthcare workers in Liberia.

The study also had some positive impact. First, we reached most of the health workforce during our study. There are approximately 300 people who work at JFK Medical Center and our study was able to test 245. Second, as part of our study, we increased awareness of hepatitis B and C. Each participant was given information about transmission, signs, symptoms and treatment. As a result, increased vaccination occurred in coordination with the JFK pharmacy staff. We discussed the hepatitis B vaccine with all patients that screened HBsAg negative and each patient was referred to the pharmacy where they could receive the vaccine at a reduced cost of approximately 750 Liberian dollars (approximately 4 US dollars). They were also given instructions for scheduling the follow-up two doses.

Furthermore, investigations such as ours have helped fuel the national campaign. Our research was performed in response to a collaborative effort with the Ministry of Health of Liberia, WHO, Clinton Health Access Initiative (CHAI), and Liberian Cancer Society, in the development of the National Cancer Policy [16]. One of the aims of the National Cancer Policy was prevention. With concerns of increasing liver cancer cases in Liberia, there was a need for in-country prevalence data of hepatitis B and C, especially in healthcare workers. And since the original findings of this paper, a national campaign has been established to target 16,000 healthcare workers for vaccination for hepatitis B in Liberia [17].

## Conclusions

Establishing a first line testing prevalence rate of 6.12% of hepatitis B and a sample prevalence of 4.89% among confirmed negative cases, along with a 0% of hepatitis C among healthcare workers in Liberia, is a significant addition to current knowledge of rates of hepatitis in Liberia. By establishing baseline prevalence rates, future interventions can now focus on increasing public health awareness, decreasing transmission, improving vaccination efforts, and treatment options. Given that there is no data on hepatitis B and C rates in Liberia our findings are important for further public health efforts focusing on at risk populations.
